# Physical activity and biological maturation: a systematic review

**DOI:** 10.1016/j.rpped.2014.11.003

**Published:** 2015-03

**Authors:** Eliane Denise Araújo Bacil, Oldemar Mazzardo, Cassiano Ricardo Rech, Rosimeide Francisco dos Santos Legnani, Wagner de Campos

**Affiliations:** a Universidade Federal do Paraná, Curitiba, PR, Brazil; b Universidade Federal de Santa Catarina, Florianópolis, SC, Brazil

**Keywords:** Puberty, Motor activity, Child, Adolescent

## Abstract

**OBJECTIVE:**

To analyze the association between physical activity (PA) and biological maturation in children and adolescents.

**DATA SOURCE:**

We performed a systematic review in April 2013 in the electronic databases of PubMed/MEDLINE, SportDiscus, Web of Science and LILACS without time restrictions. A total of 628 potentially relevant articles were identified and 10 met the inclusion criteria for this review: cross-sectional or longitudinal studies, published in Portuguese, English or Spanish, with schoolchildren aged 9-15 years old of both genders.

**DATA SYNTHESIS:**

Despite the heterogeneity of the studies, there was an inverse association between PA and biological maturation. PA decreases with increased biological and chronological age in both genders. Boys tend to be more physically active than girls; however, when controlling for biological age, the gender differences disappear. The association between PA and timing of maturation varies between the genders. Variation in the timing of biological maturation affects the tracking of PA in early adolescent girls. This review suggests that mediators (BMI, depression, low self-esteem, and concerns about body weight) can explain the association between PA and biological maturation.

**CONCLUSIONS:**

There is an association between PA and biological maturation. PA decreases with increasing biological age with no differences between genders. As for the timing of biological maturation, this association varies between genders.

## Introduction

Regular physical activity (PA) brings benefits in the short and long-term to the cardiovascular, bone, muscle and psychological health of children and adolescents.^1^^,^ 2 During childhood and adolescence, boys tend to perform more PA than girls.^3^ In both genders, PA levels decrease with age, especially during adolescence. The decrease is greater among younger girls (9-12 years) and older boys (13-16 years),^4^ and it manifests in many contexts, including active transportation, physical education classes and leisure PA, and may extend into adulthood.^5^^-^ 7

The decline in PA among adolescents seems to be more associated with biological age than with chronological age.^8^ Biological maturation is a factor that can change the pattern of PA in children and adolescents. Biological maturation refers to the progression toward the state of maturity, and can be analyzed by two components: timing and time. "Timing" is considered the moment when a given maturation event occurs. For instance, the age of menarche, the growth spurt period, the appearance of secondary sexual characteristics, among others. When identifying the timing of biological maturation of an individual, it is possible to classify this individual as early-maturing, on time or late-maturing. "Time" is the rate at which this event is expressed, i.e., how fast or slow these changes manifest themselves.^9,10^

Individuals become less physically active as they progress toward the state of maturity, regardless of chronological age.^10^ The different timing of sexual maturation and the growth spurt related to age and gender may be relevant to this decrease in PA.^8^ In girls, the onset of secondary sexual characteristics, such as breast development, may contribute to perceptions of discomfort and lower self-esteem, which favors a decreased participation in PA.^11^ Additionally, hormonal changes and changes in body composition, such as increased body fat, which are characteristic of this phase, may be related to reduced PA.^12^ In boys, the age of the early peak height velocity (PHV) can positively influence the behavior of PA due to increased muscle mass and strength, which tends to occur after the peak height velocity (PHV) point.^13^ These changes in body composition and physical fitness promote better sports performance. Individuals who regularly participate in sports during adolescence tend to perform more daily PA, and are more likely to perform PA in adulthood.^14^ The understanding of the biological maturation process influence on the practice of PA may allow the identification of adequate maturation time for intervention and behavior modification, in which the decrease in PA is more prominent in children and adolescents.

However, the association between timing of biological maturation (early, on time or late) and participation in PA is still controversial in literature. Studies have indicated that the risk of insufficient PA is twice as high in boys who matured earlier than in their peers who matured on time.^15^ However, other studies suggest that boys who mature early have significantly higher values of vigorous PA compared to boys who matured on time or late.^16^ Regarding girls, studies indicate that those who matured early perform less PA in their daily life.^17^^,^ 18 However, other studies show no such association.^16^^,^ 19

Additionally, the variation in the timing of sexual maturation may partially explain the tracking of PA.^1^^,^ 6  Tracking of PA refers to the individual's tendency to maintain the behavior over time and is generally expressed by calculating the correlation between repeated measurements of PA in the same individual.^20^ If the tracking of PA is high from childhood to adulthood, it is necessary to implement interventions since childhood and adolescence to change behavior; however, if the tracking is low, it is suggested that being inactive in childhood does not predict being inactive in adulthood, and thus intervention strategies in adult life may be more effective.^6^

The association between timing of biological maturation and participation in PA has not been systematically analyzed. There are many analytical and methodological limitations in research, the results so far seem to be inconsistent and associations are usually low. The data are limited by small sample size, the failure to consider external variables that influence this behavior and the quality of assessment of biological maturation and PA in adolescents.^8^

Therefore, the present study systematically reviewed the literature to identify studies that assessed the association between PA and biological maturation in children and adolescents. To date, no systematic review was carried out to analyze the studies associating PA and biological maturation. In order to fill this knowledge gap, the aim of this study was to review and systematize the findings of studies that investigated the association between PA and biological maturation in children and adolescents.

## Method

We carried out a systematic literature review, which identified articles describing the association between PA and biological maturation. The search was performed in April 2013, at the electronic databases of PubMed/MEDLINE, SportDiscus, Web of Science and LILACS. The combination of descriptors in English (Medical Subject Headings - MeSH) and Portuguese (Descriptors of Health Sciences - DECS) and text words were used to generate the list of citations. The research process was built specifically for each database and no limit was used in this research. The keywords were used to search in PubMed/MEDLINE, Web of Science and LILACS, and by topic in Web of Science. The survey was performed with descriptors in English and Portuguese.

The search strategy was based on a combination of four search parameters: independent variable (biological maturation), the dependent variable (PA), the population of interest (schoolchildren) and age range (9-15 years). The keywords for biological maturation were stratified in three subgroups: (i) "growth and development" [Subheading] OR "growth and development" [MeSH Terms] OR growth and development [Text Word]; (ii) "puberty" [MeSH Terms] OR puberty [Text Word]; (iii) "maturity" [Text Word] OR "maturation" [Text Word], and for PA they were: "motor activity" [MeSH Terms] OR motor activit* [Text Word] OR "exercise" [MeSH Terms] OR exercise* [Text Word]. 

Each subgroup of PA and biological maturation was used in combination with keywords for the type of sample ("students" [MeSH Terms] OR student* [Text Word]) and ("child" [MeSH Terms] OR child* [Text Word] OR "adolescent" [MeSH Terms] OR adolescent* [Text Word]) to locate potentially relevant studies. The Boolean AND operator was used to combine the four groups in the survey. The symbol (*) was used to capture all suffix variations of the root word.

Articles were selected according to the systematic review method. The period of article publication was not restricted for the search. The search in databases of electronic journals and the selection of titles, abstracts and articles were made by two researchers independently, strictly following the pre-defined inclusion and exclusion criteria. The methodological quality of each selected article was analyzed individually and then discussed by the two researchers to reach a consensus. In case of disagreement, the opinion of a third reviewer was requested. 

Inclusion criteria for the study were: (i) studies published in indexed journals; (ii) schoolchildren aged 9 to 15 years; (iii) individuals of both genders; (iv) cross-sectional and longitudinal design; (iv) language: Portuguese, English and Spanish. Exclusion criteria were: (i) sample of individuals aged under 9 and over 15 years; (ii) sample with pregnant adolescents; (iii) adolescents with hormonal diseases; (iv) adolescents with some type of syndrome. These criteria were selected in order to increase comparability between studies. 

An initial analysis was carried out based on the titles of the manuscripts. When the title and the abstract were inconclusive, i.e., when there were doubts regarding the article content, the full text was sought, to not run the risk of leaving important studies out of the systematic review. Duplicate titles were selected and the repeated title was excluded from the selection. After title selection, evaluators read the titles independently and then discussed to reach a consensus for the selection of abstracts. The same process was applied to the abstracts in order to select articles. Thus, after reviewing the titles and abstracts, all articles with full text were obtained and included if they met the inclusion criteria, and were subsequently read in full by the researchers. The references of all selected articles were assessed to identify other publications that could be included in the review.

## Results

The literature search identified 628 potentially relevant articles in the assessed databases. According to the inclusion criteria, after reading the titles and excluding 25 duplicate titles, 23 articles were selected for abstract reading. Then, after the evaluation of the abstracts, 10 articles were read in full, of which only seven were selected. The three studies were excluded due to the following reasons: one was a thesis, the second did not have the variable of interest (PA), and the third did not associate PA and biological maturation. Two studies that did not specifically contemplate the age range of 9 to 15 years, but comprised schoolchildren at these ages were included. Subsequently, three studies were found in the references of articles read in full and were added to the review. In the end, 10 articles were included in this review.^1^^,^ 5 ^,^ 6 ^,^ 17 ^,^ 21 ^-^ 26 (Table 1).

**Table 1:** Studies that investigated the association between physical activity and sexual maturation in children and adolescents.

Reference	Sample	Study design	Measure of Physical Activity	Measure of Biological Maturation	Statistical Treatment	Physical Activity and Biological Maturation Association
Gebremarian et al^1^	885 schoolchildren (11.2 years)	L	Self-reported	PDS	Multiple regression	There was no significant association
Erlandson et al^6^	187 schoolchildren (8-15 years)	L	Self-reported	APHV	Intraclass correlation coefficient	PA decreased with increase of CA and BA
Drenowatz et al^17^	268 girls (9.5-11.5 years)	C	Pedometer	APHV	ANOVA with post-hoc Tukey test and ANCOVA	EM lower PA in girls
Cumming et al^5^	185 schoolchildren (13-15 years)	L	Self-reported	PPAH	Pearson’s correlation	EM higher PA in boys and lower PA in girls
Cumming et al^24^	186 schoolchildren (13-14 years)	C	Self-reported	PPAH	Pearson’s Correlation	EM higher PA in boys
Sherar et al^25^	401 children (8-13 years)	C	Accelerometer	APHV	Two-Way ANOVA	PA decreased with increase in BA
Davison et al^21^	178 girls (11 years)	L	Self-reported and accelerometer	Tanner stage, PDS and estradiol levels.	Spearman’s correlation and structural equation modeling	EM less pleasure for PA and lower PA
Thompson et al^22^	138 children (9-18 years)	L	Self-reported	APHV	Student’s t test for unpaired samples and linear random effect model	PA decreased with increase in BA
Simon et al^26^	4320 schoolchildren (11-12 years)	C	Self-reported	PDS	Logistic and ordinal regression	EM higher PA in boys
Kemper et al^23^	200 individuals (12-22 years)	L	Structured interview	Skeletal maturation	MANOVA and ANOVA	LM higher PA

L, longitudinal; C, cross-sectional; PA, physical activity; CA, chronological age; BA, biological age; APHV, age at peak height velocity; PDS, Pubertal Development Scale; PPAH, Percentage of predicted adult height; EM, early biological maturation; LM, late biological maturation.

Table 1 shows the overall information about the 10 articles included in the systematic review. The use of different methods for evaluating PA and biological maturity was observed. Six articles were classified as longitudinal^1^^,^ 5 ^,^ 6 ^,^ 21 ^-^ 23, and four as cross-sectional studies.^17^^,^ 24 ^-^ 26

The tools used to assess PA were diverse, with the predominant use of self-reported measures.^1^^,^ 5 ^,^ 6 ^,^ 21 ^,^ 22 ^,^ 24 ^,^ 26 Other criteria used were accelerometer,^21^^,^ 25 pedometer^17^ and structured interview.^23^ The tools used to classify biological maturation also differed. The age at peak height velocity was the most often used tool,^6^^,^ 17 ^,^ 22 ^,^ 25 followed by the Pubertal Development Scale,^1^^,^ 21 ^,^ 26 percentage of predicted adult height,^5^^,^ 24 Tanner stages and analysis of estradiol levels^21^ and Skeletal Maturation.^23^

The sample ages were very heterogeneous, and four articles assessed children and adolescents,^6^^,^ 17 ^,^ 22 ^,^ 25 six assessed only adolescents (≥11 years)^1^^,^ 5 ^,^ 21 ^,^ 23 ^,^ 24 ^,^ 26, and none assessed only children. 

PA tends to decrease with increasing chronological and biological age in both genders.^6^^,^ 21 ^,^ 22 ^,^ 25 Boys tend to practice more PA than girls; however, controlling for biological age, these gender differences in PA disappear.^6^^,^ 22 ^,^ 24 ^,^ 25 For Cumming et al^24^ gender differences in PA behavior during adolescence are confounded by biological age. In this study, multivariate analysis of covariance (MANCOVA) was used to assess gender differences related to PA during leisure, controlling for biological maturation. It was observed that gender differences in relation to the practice of PA were attenuated and non-significant when biological maturation was controlled. However, in the study by Thompson et al,^22^ the authors classified individuals aged 9-18 years by chronological age groups and boys had significantly higher scores of PA than girls at 10 to 16 years. However, when classified by biological age, gender differences were not apparent, except three years before peak height velocity. 

The association between PA and timing of biological maturation (early, on time or late) is shown differently between boys and girls.^5^^,^ 6 ^,^ 17 ^,^ 23 ^,^ 24 ^,^ 26 Girls who mature early have a lower level of PA,^17^^,^ 21 and boys with advanced maturation have greater involvement in PA.^5^^,^ 23 ^,^ 24 ^,^ 26

The variation in the timing of biological maturation seems to have an effect on the PA tracking in early-maturing adolescent girls.^6^ The tracking of PA controlled by biological age was moderate to high in both genders.^6^ The articles in this review suggest mediating variables (body mass index - BMI, depression, low self-esteem and concerns with body weight) as potential explanations of the association between PA and biological maturation.^5^^,^ 17 ^,^ 21

## Discussion

### Physical activity and time of biological maturation

According to the reviewed studies, an inverse association can be observed between PA and time of sexual maturation, that is, the PA decreases with increasing biological and chronological ages in both genders.^6^^,^ 21 ^,^ 22 ^,^ 25 The process of PA reduction tends to occur at eight and ten years (preadolescence) in both boys and girls^6^^,^ 25 until approximately 15-17 years, or three to four years after the peak height velocity.^6^

Boys are usually more active than girls, but these gender differences disappear when the data are controlled by biological age.^6^^,^ 22 ^,^ 24 ^,^ 25 There is a maturational difference between boys and girls of similar chronological age. Girls, on average, mature two years before boys.^9^ It is possible, then, to attribute the lower level of PA reported by girls, when compared with boys of the same chronological age, to early maturation.^22^

It is noteworthy that not only the biological maturation is a determinant of PA practice in boys and girls, with this being a complex behavior determined by the interaction of several biological, social, economic and cultural factors. Historically and in general, girls receive a more conservative education, whether from family or society. The environment is also a factor that influences PA practice, since the neighborhood or the place of residence of the adolescent should be considered.^27^ Social support from parents and friends is also directly associated with the PA level among adolescents, and part of this association is mediated by perceptions of self-effectiveness.^28^

Two studies showed no association between PA and biological maturation. This may be due to the method of PA assessment (a single question that analyzed only PA duration)^1^ or due to small sample size (n=178).^21^ In the study by Sherar et al*,* 8 nine articles that verified the impact of biological maturation on PA were summarized, and the results were found to be inconsistent, and associations, when observed, of small magnitude, which reinforces the low methodological quality of the articles involving PA and biological maturation.

### Physical activity and timing of biological maturation

The association between PA and timing of biological maturation (early, on time or late) varies between the genders. Girls who matured earlier had lower PA levels,^17^^,^ 21 and boys with advanced maturity showed greater involvement with PA.^5^^,^ 23 ^,^ 24 ^,^ 26

The early-maturing girls can have a decrease in interest in PA practice when they experience the physical changes of adolescence, such as increased fat deposition, breast development and hip enlargement, which may hinder the motor and physiological performance, and consequently reduce disposition for physical activity. In addition to physical changes, there are also changes in interests in girls during adolescence, which tend to be similar to those of adults, such as attraction for the opposite sex and adoption of risk behaviors such as smoking, alcohol consumption and sedentary lifestyle. These behavioral changes, the increasing obligations in daily tasks, work at home and/or the transition from school to work can facilitate the reduction of PA.^24^^,^ 25

In contrast, the physical changes that occur in boys such as a gain in height, body weight, higher proportion of lean mass and the widening of shoulders, are beneficial for participation in PA, as they result in a more appropriate physical build for success in many types of PA, particularly those that emphasize speed, power and strength.^6^ Furthermore, late-maturing boys tend to perform more PA involving sports and games.

The onset of puberty may vary by up to four or five years between normal healthy boys and girls,^29^ suggesting that gender differences in biological maturation contribute to the differences in PA during childhood and adolescence.^6^ Therefore, differences in timing of biological maturation in children of the same chronological age probably have variable influence on PA participation.^6^ These differences explain why adolescent boys and girls exhibit similar rates of PA decrease, although the decrease occurs earlier in girls than in boys, and why boys are more likely to practice PA than girls of the same chronological age.

### Tracking of physical activity and its association with timing of biological maturation

During the transition from childhood to adolescence, individuals undergo physiological changes, e.g., changes in body fat, BMI and physical fitness, which influence the participation in PA.^6^ The variation in the timing of these changes in groups of the same chronological age, when PA is controlled for biological age, can affect the tracking of PA.^1^^,^ 6 ^,^ 30 Therefore, studies assess the stability of PA from childhood to late adolescence, controlling for differences in biological maturation to verify whether there is an improvement in PA tracking.^6^

In the study by Gebremariam et al*,* 1 pubertal status was not associated with PA in the baseline time and was not able to predict the level of PA and its correlates at the age of 13 in either gender. However, in the study by Erlandson et al*,* 6 the tracking of PA controlled for biological age was moderate to high in boys (r=0.44-0.60) and girls (r=0.39-0.62). The moderate-to-high tracking reinforces the assertion that PA in adults can be improved by promoting PA in childhood and adolescence. The differences between the studies may result from several factors, such as the different methods used to measure PA (differentiated self-reported questionnaires) and biological maturation (pubertal development scale and age at peak height velocity), as well as different ages of the schoolchildren in both studies.

In addition, differences in the timing of biological maturation had little effect on PA tracking in boys (r=0.30-0.46) and seem to have a greater effect on early-maturing adolescent girls (r=0.23-0.62). This suggests that maturity can be more important in PA participation for early-maturing adolescent girls. Thus, interventions, especially in females, should be implemented based on biological age groups, rather than chronological age.

### Influences on the association between PA and biological maturation

The articles in this review suggest mediating variables as potential explanatory factors of the association between PA and biological maturation.^5^^,^ 17 ^,^ 21 Adolescence is a time of great hormonal, physiological and physical changes, which are likely to influence PA patterns.^6^ These rapid changes can have psychological, emotional and behavioral effects, given that the individual is not always fully prepared for them.^5^^,^ 6 ^,^ 9

Hence, to understand how biological maturation may impact involvement in PA, it is imperative to assess the physiological, biological, psychological and social factors that mediate or moderate this association.^5^^,^ 6 The variation associated with maturation may be related to several neuroendocrine changes that trigger the onset of puberty. Physiological factors, such as the maturation of the hypothalamic-pituitary-gonadal axis, mediate the release of gonadotropins (protein hormones secreted by the posterior part of the pituitary gland and that stimulate the reproductive activity of testes and ovaries). As a result, subsequent neural reorganization and rapid changes in the maturation state and body composition may directly or indirectly affect PA.^31^ Additionally, biological (BMI)^5^^,^ 17 and psychosocial factors (depression, low self-esteem and concerns with body weight)^21^ may explain the association between PA and biological maturation.

In the study by Davison et al^21^ a more advanced pubertal development at age 11 years in adolescent girls predicts lower psychological well-being the age of 13, including depression, fear of maturation related to body weight and lower self-esteem, which in turn are predictors of lower pleasure for PA and consequently, of lower PA. These results may be due to the increase in body fat in girls with increasing maturity, resulting in reduced self-esteem and influencing their choices of PA. It is possible that early-matured girls tend to reject PA due to increased body fat and a greater concern with their body weight. However, in the study by Simon et al*,* 26 stress and psychological difficulties (hyperactivity, emotional symptoms, behavioral problems, problems in peer relationships and prosocial scale) did not mediate the association between pubertal stages and PA in boys. The differences found between studies may be due to the different measures of biological maturation (pubertal development scale, Tanner breast stage and estradiol levels), to the different psychological problems assessed and to the heterogeneity of the evaluation measures of the latter.

Among the limitations of the studies, it was observed that none of them was carried out in Latin America or in countries of low or middle income, with most studies being performed in developed countries in North America and Europe. Sample size was insufficient, especially in cross-sectional studies, not being representative of children and adolescents. The studies did not disclose the sample selection methods. Some studies used PA assessment tools without validity and reliability tests to demonstrate their legitimacy. It is also important to emphasize the difficulty to compare studies by the different measurement methods used to assess PA levels and biological maturation. 

## Final considerations

Based on this review, scientific evidence suggests that PA is inversely associated with biological maturation. PA tends to decrease with increasing chronological and biological age in both genders. Girls tend to perform less PA than boys in their daily routines; however, after controlling for biological age, gender differences in PA disappear. The association between PA and timing of biological maturation varies between the genders. Girls who mature early have a lower level of PA, and boys with advanced maturation show greater involvement in PA. The differences in biological maturation timing seem to have greater effect on PA participation in early-maturing girls. The tracking of PA controlled for biological age was moderate to high in both genders, suggesting that PA should be encouraged since childhood and adolescence. Studies indicate mediating variables as potential explanatory factors of the association between PA and biological maturation.

Considering these results, it can be observed that biological maturation seems to have an important role in the decrease of PA in adolescents. It is recommended that health-promoting efforts in childhood and adolescence, aiming at encouraging the practice of PA, should not take into account only the chronological age of individuals, but also maturational changes responsible for behavioral changes in children and adolescents. We emphasize the need for further studies to evaluate the association between PA and biological maturation, as the number of articles found was scarce. However, it is important to standardize the criteria to determine PA and biological maturation in children and adolescents in order to facilitate the comparison between studies.
