# Supplementary material for: Physical activity and biological maturation: a systematic review
Source: Rev Paul Pediatr. 2015 Mar;33(1):114–21. doi: 10.1016/j.rpped.2014.11.003 (PMC4436963; doi:10.1016/j.rpped.2014.11.003)
Supplement: Translation

# Translation: Atividade física e maturação biológica: uma revisão sistemática

**Authors:** Eliane Denise Araújo Bacil, Oldemar Mazzardo, Cassiano Ricardo Rech, Rosimeide Francisco dos Santos Legnani, Wagner de Campos

**Affiliations:** a Universidade Federal do Paraná (UFPR), Curitiba, PR, Brasil; b Universidade Federal de Santa Catarina (UFSC), Florianópolis, SC, Brasil

**Keywords:** Puberdade, Atividade motora, Crianças, Adolescentes

## Abstract

**OBJETIVO:**

Analisar a associação entre atividade física (AF) e maturação biológica em crianças e adolescentes.

**FONTES DE DADOS:**

Fez-se uma revisão sistemática em abril de 2013 nos bancos de periódicos eletrônicos da PubMed/Medline, SportDiscus, Web of Science e Lilacs, sem restrição de período. Foram identificados 628 artigos potencialmente relevantes e dez preencheram os critérios de inclusão, ou seja, eram estudos transversais ou longitudinais, publicados em português, inglês ou espanhol e envolviam escolares de nove a 15 anos, de ambos os sexos.

**SÍNTESE DOS DADOS:**

Apesar da heterogeneidade dos estudos, foi possível identificar uma associação inversa entre AF e maturação biológica. A AF diminui com o aumento da idade cronológica e biológica em ambos os sexos. Os meninos tendem a praticar mais AF do que as meninas. Contudo, controlado o efeito da idade biológica, esdas diferenças entre sexos desaparecem. A associação entre AF e timing da maturação biológica varia entre os sexos. A variação no *timing* da maturação biológica parece ter efeito no *tracking* da AF em meninas adolescentes precoces. Os artigos desta revisão sugerem variáveis mediadoras (IMC, depressão, baixa autoestima e preocupações com o peso corporal) como potenciais explicativos da associação entre AF e maturação biológica.

**CONCLUSÕES:**

Observou-se que existe uma relação inversa entre AF e maturação biológica. Com relação ao tempo da maturação biológica, a AF diminui com o aumento da idade biológica e não apresenta diferenças entre os sexos e com relação ao *timing* da maturação biológica essa relação varia entre os sexos.

## Introdução

A atividade física (AF) regular proporciona benefícios em curto e longo prazo à saúde cardiovascular, óssea, muscular e psicológica de crianças e adolescentes.^1^ ^and^ 2 Durante a infância e a adolescência, os meninos tendem a fazer mais AF do que as meninas.^3^ Em ambos os sexos, os níveis de AF diminuem com a idade, especialmente a partir da adolescência. O declínio é maior nas meninas de idades mais jovens (nove a 12 anos) e nos meninos em idades mais avançadas (13 a 16 anos)^4^ e se manifesta em muitos contextos, como transporte ativo, aulas de educação física e AF de lazer e pode se estender até a idade adulta.^5^^,^ 6 ^and^ 7

O declínio da AF no adolescente parece ser mais associado com a idade biológica do que com a idade cronológica.^8^ A maturação biológica é um fator que pode alterar o padrão de AF em crianças e adolescentes. A maturação biológica se refere à progressão em direção ao estado de maturidade e pode ser analisada por dois componentes: *timing* e tempo. *Timing* é considerado o momento em que ocorre um dado evento maturacional. Por exemplo, a idade da menarca, o período de estirão do crescimento, o aparecimento das características sexuais secundárias, entre outros. Ao identificar o *timing* da maturação biológica de um indivíduo, é possível classificá-lo em maturado precoce, no tempo ou tardio. Tempo é o ritmo com que esse evento se manifesta, ou seja, o quão lentas ou rápidas essas alterações se manifestam. 9 ^and^ 10

Os indivíduos tornam-se menos ativos fisicamente à medida que o progresso em direção ao estado de maturidade evolui, independentemente da idade cronológica.^10^ O diferente *timing* da maturação sexual e o surto de crescimento relativo à idade e sexo podem ser relevantes para esse declínio da AF. 8 Em meninas, o início do aparecimento das características sexuais secundárias, como o desenvolvimento mamário, pode contribuir para percepções de desconforto e menor autoestima, o que favorece uma menor participação em AF. 11 Além disso, mudanças hormonais e alterações na composição corporal, como o aumento de gordura corporal característicos dessa fase, podem ser relacionados ao declínio da AF. 12 Em meninos, a idade do pico de velocidade de altura (IPVA) precoce pode influenciar positivamente o comportamento de AF devido ao aumento da massa muscular e da força que tende a ocorrer depois do pico de velocidade de altura (PVA). 13 Essas modificações de composição corporal e aptidão física favorecem um melhor desempenho esportivo. Indivíduos que participam de esportes regularmente durante a adolescência tendem a fazer mais AF diária e são mais propensos a fazer AF na idade adulta. 14 Compreender a influência do processo de maturação biológica na prática de AF pode possibilitar a identificação de momentos maturacionais adequados para intervenção e modificação de comportamento nos quais a diminuição da AF é mais proeminente em crianças e adolescentes.

Contudo, ainda é controversa, na literatura, a relação entre *timing* da maturação biológica (precoce, no tempo ou tardio) e a participação na AF. Estudos apontam que o risco de AF insuficiente é duas vezes maior em meninos que maturam precocemente do que seus pares que maturam no tempo. 15 No entanto, outros estudos apontam que os meninos que maturam precocemente apresentam significativamente maiores valores de AF vigorosa comparados com meninos desenvolvidos no tempo ou tardiamente. 16 Com relação às meninas, os estudos indicam que aquelas maturadas precocemente fazem menos AF no seu cotidiano. 17 ^and^ 18 Contudo, outros estudos não apresentam tal associação. 16 ^and^ 19

Além disso, a variação no *timing* da maturação biológica pode explicar parcialmente o *tracking* da AF. 1 ^and^ 6 *Tracking* da AF refere-se à tendência do indivíduo de manter o comportamento ao longo do tempo, expresso, em geral, pelo cálculo da correlação entre medidas repetidas de AF no mesmo indivíduo. 20 Se o *tracking* da AF é alto da infância até a idade adulta, é necessário implementar intervenções desde a infância e adolescência para mudança de comportamento; entretanto, se o *tracking* é baixo, sugere-se que ser inativo na infância não prediz ser inativo na idade adulta e, dessa forma, estratégias de intervenção na vida adulta podem ser mais efetivas. 6

A relação entre *timing* da maturação biológica e a participação em AF não tem sido sistematicamente analisada. Existem muitas limitações analíticas e metodológicas em pesquisas, os resultados até agora parecem ser inconsistentes e as associações são geralmente baixas. Os dados são limitados pelo tamanho pequeno da amostra, pela não consideração de variáveis externas que influenciam esse comportamento e pela qualidade da avaliação da maturação biológica e AF em adolescentes. 8

Desse modo, o presente estudo revisou sistematicamente a literatura para identificar estudos que analisaram a relação entre AF e maturação biológica em crianças e adolescentes. Até o momento nenhuma revisão sistemática foi conduzida com a finalidade de analisar os estudos que associam AF e maturação biológica. Com o intuito de preencher essa lacuna do conhecimento, o objetivo deste estudo foi revisar e sistematizar os achados de estudos que investigaram a relação entre AF e maturação biológica em crianças e adolescentes.

## Método

Fez-se uma revisão sistemática da literatura, a qual identificou artigos que descrevessem a associação entre AF e maturação biológica. A busca foi feita em abril de 2013, nas bases de periódicos eletrônicos PubMed/Medline, SportDiscus, Web of Science e Lilacs.

A combinação dos descritores em inglês (Medical Subject Headings-[MeSH]) e em português (Descritores de Ciências da Saúde - Decs) e palavras de texto foram usadas para gerar a lista de citações. O processo de pesquisa foi construído especificamente para cada base de periódicos e nenhum limite foi usado nessas pesquisas. As palavras-chaves foram usadas para busca no PubMed/Medline, Web of Science e Lilacs e por tópico na Web of Science. A pesquisa foi feita com descritores em inglês e português.

A estratégia de pesquisa baseou-se numa combinação de quatro parâmetros: variável independente (maturação biológica), variável dependente (AF), população de interesse (escolares) e faixa etária (nove a 15 anos). As palavras-chaves para maturação biológica foram estratificadas em três subgrupos: (i) growth and development [Subheading] OR growth and development [MeSH Terms] OR growth and development [Text Word]; (ii) puberty [MeSH Terms] OR puberty [Text Word]; (iii) maturity [Text Word] OR maturation [Text Word] e para AF foram: motor activity [MeSH Terms] OR motor activit* [Text Word] OR exercise [MeSH Terms] OR exercise* [Text Word].

Cada subgrupo de AF e maturação biológica foi usado em combinação com palavras-chave para o tipo de amostra (students [MeSHTerms] OR student* [Text Word]) e (child [MeSHTerms] OR child* [Text Word] OR adolescent [MeSHTerms] OR adolescent* [Text Word]) para localizar estudos potencialmente relevantes. O operador boleano AND foi usado para combinar os quatro grupos na pesquisa. O símbolo (*) foi usado para capturar todas as variações do sufixo da palavra raiz.

Os artigos foram selecionados de acordo com o método de revisão sistemática. O período de publicação dos artigos não foi delimitado para a busca. A busca nas bases de periódicos eletrônicos e a seleção de títulos, resumos e artigos foram feitas por dois pesquisadores de forma independente, que obedeceram rigorosamente aos critérios de inclusão e exclusão definidos. A qualidade metodológica de cada artigo selecionado era analisada individualmente e posteriormente discutida entre os dois pesquisadores até alcançar um consenso. Em caso de desacordo a opinião de um terceiro avaliador foi solicitada.

Os critérios de inclusão para o estudo foram: (i) estudos publicados em revistas indexadas; (ii) escolares de nove a 15 anos; (iii) indivíduos de ambos os sexos; (iv) delineamento transversal e longitudinal; (iv) idioma: português, inglês e espanhol. Os critérios de exclusão foram: (i) amostra com indivíduos com menos de nove e mais de 15 anos; (ii) amostra com adolescentes grávidas; (iii) adolescentes que apresentavam doenças hormonais; (iv) adolescentes com algum tipo de síndrome. Esses critérios foram selecionados de modo a aumentar a comparabilidade entre os estudos.

Uma análise inicial foi feita com base nos títulos dos manuscritos. Quando o título e o resumo não eram esclarecedores, ou seja, quando se tinham dúvidas com relação ao conteúdo do artigo, buscava-se o artigo na íntegra para não correr o risco de deixar estudos importantes fora da revisão sistemática. Os títulos duplicados eram selecionados e o título repetido excluído da seleção. Após a seleção dos títulos, os avaliadores liam os títulos de forma independente e posteriormente discutiam até chegar a um consenso para a seleção dos resumos. O mesmo processo foi feito com os resumos para a seleção dos artigos. Assim, após a análise dos títulos e resumos, todos os artigos com texto na íntegra foram obtidos e incluídos se atendiam aos critérios de inclusão e posteriormente foram lidos na íntegra pelos pesquisadores. As referências de todos os artigos selecionados foram examinadas para identificar outras publicações que poderiam ser incluídas na revisão.

## Resultados

A pesquisa bibliográfica identificou 628 artigos potencialmente relevantes nas bases de periódicos investigadas. De acordo com os critérios de inclusão, após a leitura dos títulos e a exclusão de 25 títulos de artigos duplicados, 23 artigos foram selecionados para a leitura dos resumos. Em seguida, após a avaliação dos resumos, 10 artigos foram lidos na íntegra, dos quais somente sete foram selecionados. Os três estudos foram excluídos pelos seguintes motivos: um era tese, outro não apresentava a variável de interesse (AF) e um não associava AF e maturação biológica. Foram incluídos dois estudos que não contemplavam especificamente a faixa etária dos nove aos 15 anos, mas continham alunos nessas idades. Posteriormente, três estudos foram localizados nas referências dos artigos lidos e adicionados à revisão. No fim, 10 artigos participaram deste estudo^1^^,^ 5 ^,^ 6 ^,^ 17 ^,^ 21 ^,^ 22 ^,^ 23 ^,^ 24 ^,^ 25 ^and^ 26 (fig. 1).

**Figura 1:** Fluxograma do processo de seleção do estudo.

Na tabela 1, são apresentadas as informações gerais sobre os 10 artigos incluídos na revisão sistemática. Observou-se o uso de diferentes métodos para a avaliar a AF e a maturação biológica. Seis artigos foram classificados como longitudinais^1^^,^ 5 ^,^ 6 ^,^ 21 ^,^ 22 ^and^ 23 e quatro como transversais.^17^^,^ 24 ^,^ 25 ^and^ 26

**Tabela 1:** Estudos que investigaram associação entre atividade física e maturação biológica em crianças e adolescentes

Referência	Amostra	Design do estudo	Medida de atividade física	Medida de maturação biológica	Tratamento estatístico	Associação atividade física e maturação biológica
Gebremarian et al.^1^	885 escolares (11,2 anos)	L	Autorreportado	EDP	Regressão múltipla	Não houve associação significativa.
Erlandson et al.^6^	187 escolares (8-15 anos)	L	Autorreportado	IPVA	Coeficiente de correlação intraclasse	AF diminuiu com o aumento da IC e IB.
Drenowatz et al.^17^	268 meninas (9,5- 11,5 anos)	T	Pedômetro	IPVA	Anova com teste *poshoc* de Tukey e Ancova	MP menor AF em meninas.
Cumming et al.^5^	185 escolares (13-15 anos)	L	Autorreportado	PAAP	correlação de Pearson	MP maior AF em meninos e menor AF em meninas.
Cumming et al.^24^	186 escolares (13-14 anos)	T	Autorreportado	PAAP	Correlação de Pearson	MP maior AF em meninos.
Sherar et al.^25^	401 crianças (8-13 anos)	T	Acelerômetro	IPVA	Anova two-way	A AF diminuiu com o aumento da IB.
Davison et al.^21^	178 meninas (11 anos)	L	Autorreportado e acelerômetro	Estágios de Tanner, EDP e níveis de estradiol.	Correlação de Spearman e modelagem de equações estruturais	MP menor prazer para AF e menor AF.
Thompson et al.^22^	138 crianças (9-18 anos)	L	Autorreportado	IPVA	Teste *t* de Student não pareado e Modelagem de efeito linear randomizado	A AF diminuiu com o aumento da IB.
Simon et al.^26^	4320 escolares (11-12 anos)	T	Autorreportado	EDP	Regressão logística e ordinal	MP maior AF em meninos.
Kemper et al.^23^	200 indivíduos (12-22 anos)	L	Entrevista estruturada	Maturação esquelética	Manova e Anova	MT maior AF

L, longitudinal; T, transversal; AF, atividade física; IC, idade cronológica; IB, idade biológica; IPVA, idade do pico de velocidade de altura; EDP, escala de desenvolvimento puberal; PAAP, percentual de altura adulta predita; MP, maturação biológica precoce; MT, maturação biológica tardia.

Os instrumentos usados para avaliar a AF foram variados, predominou o emprego de medidas autorreportadas.^1^^,^ 5 ^,^ 6 ^,^ 21 ^,^ 22 ^,^ 24 ^and^ 26 Outros critérios empregados foram acelerometria,^21^ ^and^ 25 pedômetro^17^ e entrevista estruturada.^23^ Os instrumentos usados para classificar a maturação biológica também diferiram. A idade do pico de velocidade de altura foi a ferramenta mais usada,^6^^,^ 17 ^,^ 22 ^and^ 25 seguida da escala de desenvolvimento puberal,^1^^,^ 21 ^and^ 26 percentual de altura adulta predita,^5^ ^and^ 24 estágios de Tanner e análise dos níveis de estradiol^21^ e maturação esquelética.^23^

As idades das amostras foram bastante heterogêneas, quatro artigos avaliaram crianças e adolescentes,^6^^,^ 17 ^,^ 22 ^and^ 25 seis avaliaram somente adolescentes (≥11anos)^1^^,^ 5 ^,^ 21 ^,^ 23 ^,^ 24 ^and^ 26 e nenhum avaliou somente crianças.

A AF tende a diminuir com o aumento da idade cronológica e biológica em ambos os sexos.^6^^,^ 21 ^,^ 22 ^and^ 25 Os meninos tendem a praticar mais AF do que as meninas, no entanto, controlado o efeito da idade biológica, essas diferenças entre sexos na AF desaparecem.^6^^,^ 22 ^,^ 24 ^and^ 25 Para Cumming et al.^24^ as diferenças entre sexos no comportamento da AF durante a adolescência são confundidas pela idade biológica. Neste estudo, a análise de covariância multivariada (Mancova) foi usada para examinar as diferenças relacionadas ao sexo na AF de lazer, controladas pela maturação biológica. Verificou-se que as diferenças entre sexos em relação à prática de AF foram atenuadas e não significantes quando a maturação biológica foi controlada. No entanto, no estudo de Thompson et al.^22^ os autores classificaram os indivíduos de nove a 18 anos por grupos de idade cronológica e os meninos apresentaram significativamente maiores escores de AF do que as meninas dos 10 aos 16 anos. Entretanto, quando classificados pela idade biológica, as diferenças entre sexos não eram aparentes, exceto três anos antes do pico de velocidade de altura.

A relação entre AF e o *timing* da maturação biológica (precoce, no tempo e tardio) é apresentada de forma diferenciada entre meninos e meninas. 5 ^,^ 6 ^,^ 17 ^,^ 23 ^,^ 24 ^and^ 26 As meninas que maturam precocemente apresentam menor nível de AF, 17 ^and^ 21 bem como os meninos com maturação avançada apresentam maior envolvimento em AF. 5 ^,^ 23 ^,^ 24 ^and^ 26

A variação no *timing* da maturação biológica parece ter efeito no *tracking* da AF em meninas adolescentes precoces. 6 O *tracking* da AF controlado pela idade biológica foi moderado para alto em ambos os sexos. 6 Os artigos desta revisão sugerem variáveis mediadoras (índice de massa corporal (IMC), depressão, baixa autoestima e preocupações com o peso corporal) como potenciais explicativos da associação entre AF e maturação biológica. 5 ^,^ 17 ^and^ 21

## Discussão

### Atividade física e tempo da maturação biológica

De acordo com os estudos revisados, pode-se observar uma associação inversa entre AF e tempo de maturação biológica, ou seja, a AF diminui com o aumento da idade cronológica e biológica em ambos os sexos.^6^^,^ 21 ^,^ 22 ^and^ 25 O processo de diminuição da AF tende a ocorrer a partir dos oito e dez anos (pré-adolescência) tanto em meninos quanto em meninas^6^ ^and^ 25 até aproximadamente os 15-17 anos ou três a quatro anos depois do pico de velocidade de altura.^6^

Os meninos geralmente são mais ativos do que as meninas, mas essas diferenças entre os sexos desaparecem quando os dados são controlados pela idade biológica.^6^^,^ 22 ^,^ 24 ^and^ 25 Existe uma diferença maturacional entre meninos e meninas de similar idade cronológica. As meninas, em média, maturam dois anos antes dos meninos.^9^ É possível então atribuir o menor nível de AF relatado pelas meninas, quando comparado com o de meninos de mesma idade cronológica, à maturação precoce.^22^

É importante destacar que não somente a maturação biológica é determinante da prática de AF em meninos e meninas, esse é um comportamento complexo determinado pela interação de diversos fatores biológicos, sociais, econômicos e culturais. Historicamente e de forma geral, as meninas recebem uma educação mais conservadora, seja de sua família ou da sociedade. O ambiente é também um fator influenciador da prática de AF, uma vez que a vizinhança e o local de moradia do adolescente devem ser considerados.^27^ O apoio social dos pais e amigos também está diretamente associado com o nível de AF entre adolescentes e parte dessa associação é mediada pela percepção de autoeficácia.^28^

Dois estudos não apresentaram associação entre AF e maturação biológica. Isso pode ter ocorrido devido ao método de avaliação da AF (uma única questão, a que analisou somente duração da AF)^1^ ou devido ao pequeno tamanho da amostra (n = 178).^21^ No estudo de Sherar et al.,^8^ foram sumarizados nove artigos que verificaram o impacto da maturação biológica na AF, os resultados encontrados foram inconsistentes e as associações, quando notadas, de pequena magnitude, o que reforça a baixa qualidade metodológica dos artigos que envolvem AF e maturação biológica.

### Atividade física e timing da maturação biológica

A associação entre AF e *timing* da maturação biológica (precoce, no tempo ou tardio) varia entre os sexos. As meninas que maturam precocemente apresentaram menor nível de AF, 17 ^and^ 21 bem como os meninos com maturação avançada apresentaram maior envolvimento em AF. 5 ^,^ 23 ^,^ 24 ^and^ 26

As meninas maturadas precocemente podem diminuir o interesse pela prática de AF quando experienciam mudanças físicas próprias da adolescência, tais como: aumento do depósito de gordura, desenvolvimento mamário e alargamento dos quadris, que podem dificultar o desempenho motor e fisiológico e, consequentemente, reduzir a disposição para a atividade física. Além de mudanças físicas, ocorrem também mudanças de interesses em meninas a partir da adolescência, que tendem a ser semelhantes aos dos adultos, como: atração pelo sexo oposto e adoção de comportamentos de risco, como consumo de fumo e álcool e estilo de vida sedentário. Essas mudanças de comportamento, o aumento das obrigações das tarefas diárias, o trabalho em casa e ou a transição da escola para o trabalho podem favorecer a diminuição de AF.^24^ ^and^ 25

Ao contrário, as mudanças físicas que ocorrem em meninos, como ganho em altura, peso corporal, maior proporção de massa magra e alargamento dos ombros, são benéficas para a participação em AF, pois resultam em um aporte físico mais adequado para o sucesso em muitas formas de AF, particularmente aquelas que enfatizam velocidade, potência e força.^6^ Além disso, meninos maturados tardiamente tendem a fazer mais AF que envolva jogos e brincadeiras.

O aparecimento da puberdade pode variar em até quatro ou cinco anos entre meninos e meninas saudáveis normais,^29^ o que sugere que as diferenças entre sexos na maturação biológica contribuem para as diferenças na AF durante a infância e adolescência.^6^ Dessa forma, as diferenças no *timing* da maturação biológica em crianças de mesma idade cronológica, provavelmente, apresentam influência variável na participação em AF. 6 Essas diferenças explicam por que meninos e meninas adolescentes exibem similares taxas de declínio da AF, apesar de o declínio ocorrer mais cedo nas meninas do que nos meninos e porque os meninos são mais propensos a praticar AF do que as meninas de mesma idade cronológica.

### Tracking da atividade física e sua relação com o timing da maturação biológica

Durante a transição da infância para a adolescência, os indivíduos passam por mudanças fisiológicas, como, por exemplo, alterações na gordura corporal, IMC e aptidão física, que influenciam na participação de AF.^6^ A variação no *timing* dessas mudanças em grupos de mesma idade cronológica, quando a AF é controlada pela idade biológica, pode afetar o *tracking* da AF. 1 ^,^ 6 ^and^ 30 Dessa forma, estudos examinam a estabilidade da AF ao longo da infância até o fim da adolescência e controlam diferenças na maturação biológica para verificar se há uma melhoria no *tracking* da AF. 6

No estudo de Gebremariam et al.,^1^ o status puberal não foi associado com AF no tempo de base e não foi capaz de predizer o nível de AF e seus correlatos na idade de 13 anos em qualquer um dos gêneros. No entanto, no estudo de Erlandson et al.^6^ o *tracking* da AF controlado pela idade biológica foi moderado a alto em meninos (r = 0,44-0,60) e meninas (r = 0,39-0,62). O *tracking* moderado a alto reforça a afirmação de que a AF no adulto pode ser melhorada por meio da promoção da AF a partir da infância e adolescência. As diferenças entre os estudos podem resultar de diversos fatores, como os diferentes métodos usados para medir AF (questionários autorreportados diferenciados) e a maturação biológica (escala de desenvolvimento puberal e idade do pico de velocidade de altura), bem como as diferentes idades dos escolares nos dois estudos.

Além disso, as diferenças no *timing* da maturação biológica apresentaram pouco efeito no *tracking* da AF em meninos (r = 0,30-0,46) e parecem ter um efeito maior em meninas adolescentes precoces (r = 0,23-0,62). Isso sugere que a maturidade pode ser mais importante na participação de AF em meninas adolescentes precoces. Assim, intervenções, principalmente em meninas, devem ser implementadas com base em grupos de idade biológica, mais do que em idade cronológica.

### Influências na relação entre atividade física e maturação biológica

Os artigos desta revisão sugerem variáveis mediadoras como potenciais explicativas da associação entre AF e maturação biológica.^5^^,^ 17 ^and^ 21 A adolescência é um período de grandes mudanças hormonais, fisiológicas e físicas, as quais provavelmente influenciam os padrões de AF.^6^ Essas mudanças rápidas podem gerar efeitos psicológicos, emocionais e comportamentais, dado que o indivíduo nem sempre está plenamente preparado para tal.^5^^,^ 6 ^and^ 9

Assim, para entender como a maturação biológica pode impactar o envolvimento em AF é imperativo examinar os fatores fisiológicos, biológicos, psicológicos e sociais que mediam ou moderam essa relação.^5^ ^and^ 6 A variação associada à maturidade pode ser relacionada a diversas mudanças neuroendócrinas que provocam o início da puberdade. Fatores fisiológicos como a maturação do eixo hipotalâmico-hipófise-gonadal mediam a liberação de gonadotropinas (hormônios proteicos secretados pela parte posterior da hipófise e que estimulam a atividade reprodutiva dos testículos e dos ovários). Com isso, a subsequente reorganização neural e as mudanças rápidas no estado de maturidade e na composição corporal podem direta ou indiretamente influenciar a atividade física.^8^ Além disso, fatores biológicos (IMC)^5^ ^and^ 17 e psicossociais (depressão, baixa autoestima e preocupações com o peso corporal)^21^ podem explicar a ligação entre AF e maturação biológica.

No estudo de Davison et al.^21^ o desenvolvimento puberal mais avançado na idade de 11 anos em meninas adolescentes prediz menor bem-estar psicológico na idade de 13 anos, incluindo depressão, medo da maturidade relacionado ao peso corporal e menor autoestima, o que, por sua vez, são fatores preditores de menor prazer para AF e, consequentemente, menor AF. Esses resultados podem ser decorrentes do aumento da gordura corporal em meninas com o aumento da maturidade, o que ocasiona uma menor autoestima e influencia suas escolhas de prática de AF. É possível que meninas maturadas precocemente tendam a rejeitar AF devido à maior gordura corporal e a uma maior preocupação com o seu peso corporal. No entanto, no estudo de Simon et al.^26^ o estresse e as dificuldades psicológicas (hiperatividade, sintomas emocionais, problemas de conduta, problemas de relacionamento com colegas e escala pró-social) não mediam a relação entre os estágios puberais e a AF em meninos. As diferenças encontradas entre os estudos podem ser decorrentes das diferentes medidas de maturação biológica (escala de desenvolvimento puberal, estágio mamário de Tanner e níveis de estradiol), dos diversos problemas psicológicos avaliados e da heterogeneidades de medidas de avaliação desses.

Dentre as limitações dos estudos, observou-se que nenhum deles foi feito na América Latina ou em países de renda baixa ou média, a maioria das pesquisas foi feita em países desenvolvidos da América do Norte e da Europa. O tamanho da amostra foi insuficiente, principalmente em estudos de delineamento transversal, não foi representativo de crianças e adolescentes. As pesquisas não apresentaram os métodos de seleção das amostras. Alguns estudos apresentavam instrumentos de avaliação da AF sem testes de validade e fidedignidade para demonstrar sua legitimidade. É importante também destacar a dificuldade de comparar estudos pelos diferentes métodos de mensuração usados para avaliar os níveis de AF e maturação biológica.

## Considerações finais

Com base nesta revisão, evidências científicas apontam que a AF está associada de forma inversa com a maturação biológica. A AF tende a diminuir com o aumento da idade cronológica e biológica em ambos os sexos. As meninas tendem a fazer menos AF do que os meninos no seu dia a dia. Contudo, ao controlar a idade biológica, as diferenças entre sexos na AF desaparecem. A associação entre AF e *timing* da maturação biológica varia entre os sexos. As meninas que maturam precocemente apresentam menor nível de AF e os meninos com maturação avançada apresentam maior envolvimento em AF. As diferenças no *timing* da maturação biológica parecem ter maior efeito na participação de AF em meninas que maturam precocemente. O *tracking* da AF controlado pela idade biológica foi moderado para alto em ambos os sexos, o que sugere que a AF deva ser estimulada desde a infância e a adolescência. Os estudos indicam variáveis mediadoras como potenciais explicativos da associação entre AF e maturação biológica.

De posse desses resultados, a maturação biológica parece ter papel proeminente na diminuição da AF em adolescentes. Recomenda-se que esforços na promoção da saúde na infância e adolescência com vistas ao incentivo à prática de AF não levem em consideração somente a idade cronológica dos indivíduos, mas também as mudanças maturacionais responsáveis pela mudança de comportamento em crianças e adolescentes. Ressalta-se a necessidade de novos estudos que avaliem a associação entre AF e maturação biológica, já que o número de artigos encontrados foi escasso. Contudo, é importante padronizar critérios para determinar a AF e a maturação biológica em crianças e adolescentes para facilitar a comparação entre os estudos.
